# The HIV/AIDS pandemic will not end by the year 2030 in low and middle income countries

**DOI:** 10.11604/pamj.2019.32.67.17580

**Published:** 2019-02-07

**Authors:** Luchuo Engelbert Bain, Elvis Enowbeyang Tarkang, Ikenna Desmond Ebuenyi, Raoul Kamadjeu

**Affiliations:** 1 The Pan African Medical Journal, Nairobi, Kenya; 2 Athena Institute for Research on Innovation and Communication in Health and Life Sciences, Vrije Universiteit Amsterdam, The Netherlands; 3 School of Public Health, University of Health and Allied Sciences PMB 31 Ho, Ghana; 4 HIV/AIDS Prevention Research Network Cameroon PO Box 36 Kumba, Cameroon

**Keywords:** HIV, stop, 2030, low and middle-income

## Abstract

The recent Lancet Commission-International AIDS Society report: Advancing Global health and strengthening the HIV response in the Era of the Sustainable Development Goals; clearly highlights the fact that the world is NOT on track in ending the AIDS pandemic by 2030. Emphasis on massive and early diagnosis and placement on Combined Anti- Retroviral Therapy (cART) remain key cornerstones in reaching these goals. Effective viral load informed care remains very promising in reducing drug resistance, and improving outcomes in infected persons. The authors argue that the current funding trends, management paradigms, research agendas, data collection and information system models, as well as the overall appreciation of the evolution of the pandemic in low and middle- income countries, lead to a logical conclusion that this pandemic will not end, especially in these countries by 2030. Major action areas are proposed for policy makers and researchers for appreciation and action.

## Editorial

The recent Lancet Commission-International AIDS Society report: Advancing Global health and Strengthening the HIV response in the Era of the Sustainable Development Goals; clearly highlights the fact that the world is NOT on track in ending the AIDS pandemic by 2030 [1]. Many persons in developing countries still do not know their HIV status [1, 2]. Emphasis on massive and early diagnosis and placement on Combined Anti- Retroviral Therapy (cART) remain key cornerstones in reaching these goals. The test and treat approach has heightened hopes in encouraging self- testing, as a possible tool, which is almost stigma free, and allows hard to reach groups (e.g Men Who Have Sex with men; MSM, Intra venous Drug Users; IDUs) to know their HIV serological status [3]. It has been argued elsewhere that this approach must only complement clinic based testing, as linkage to care, sustainability concerns, clear self-testing national policies, and quality assurance challenges still constitute understudied and underexplored barriers, especially in the most infected and affected countries of the world [4-6]. In low and middle- income countries (LMICs), stigma, discrimination and access to key populations (MSM, transgender persons, sex workers, IDUs, and prisoners) might constitute a key impediment in preventing new infections. For instance, key populations accounted for over 47% of new infections in 2017 [7]. The risk of acquiring HIV is 27 times higher among men who have sex with men; 23 times higher among people who inject drugs; 13 times higher for female sex workers; 12 times higher for transgender women [8]. Key populations stand to constitute a breeding ground for a new cycle of new infections in the shadows. However, the world has been too ambitious in forgetting so early that HIV related stigma is real, serious and remains a major issue of concern [7-10]. This parameter must be provided the real attention it deserves.

Many countries are still unable to maintain a reasonable and effective supply chains for cART [11]. There is indisputable evidence that placement of People Living with HIV (PLHIV) on cART is key in breaking the transmission chain [12, 13]. This gap makes attaining 90-90-90 UNAIDS goals unrealistic, when it comes to breaking the transmission chain, as well as overcoming the barrier of drug resistance. The challenge regarding resistance to first line anti-retroviral therapy is particularly worrisome. It is an illusion regarding ending the pandemic, without making 2^nd^ and 3^rd^ line drugs available, and properly training clinicians in identifying on clinical and paraclinical grounds, treatment failure, and properly switching to appropriate 2^nd^ and 3^rd^ line drugs when indicated, and on time. We welcome efforts made lately regarding vulgarization of the viral load informed care in current HIV medicine on a global scale [1]. However, without properly ensuring adherence to available medications, proper timely switching from 1st to 2^nd^/3^rd^ line drugs, and a constant supply chain, these efforts might be diluted, and might not yield expected results.

HIV in this era is a chronic disease. The success obtained with cART in making people live longer has unfortunately increased the Non Communicable Disease burden, already challenging health systems in LMICs: HIV induced cardiometabolic disease (Heart disease, stroke, dyslipidemias, glucose intolerance to overt HIV-cART induced diabetes). Special efforts have to be made in creating appropriate chronic disease management paradigms in PLHIV [14-16]. As at now, are we ready to welcome management of HIV as a chronic disease? Primary health care models have to adapt to accommodate HIV as chronic disease in its care continuum. A lot is still not known on health outcomes in PLHIV on cART, especially from the most infected and affected regions of the world like Africa [17, 18]. Most studies are from high income countries. High income countries have more stable supply chains, and the drugs used in these countries are very different. Applying findings from the latter, to the former, in disease management is almost irrational. Cohort studies including children and women are almost inexistent in Africa [18]. It is important to note that over 1.8 million new infections were recorded worldwide for the year 2017 alone [8]. As efforts continue in identifying effective drugs with fewer side effects, the HIV vaccine research agenda must remain a priority within the HIV scientific community. Holistic and context specific approaches are highly needed to fight the emerging pandemic of drug resistance. There is lack of quality data, especially in LMICs, which makes monitoring of the pandemic very problematic [17, 18]. Funders and governments must specifically include quality data collection and information schemes in their health system strengthening agendas of countries as a whole, and for HIV in particular. Dependence on foreign aid and imported management models will not be helpful in accelerating the ending of this pandemic at diagnostic, treatment and monitoring levels. The current funding trends are insufficient to end the HIV pandemic by 2030 in LIMCs [1, 19].

The World Health Organization (WHO) is mobilizing material and financial resources for its 2017-2021 plans to support a coordinated international effort to prevent, monitor and respond to the emergence of HIV drug resistance, and to strengthen country efforts to achieve the global HIV targets [19]. If the momentum in funding the fight against the HIV-AIDS pandemic maintains the current gear, it might be too idle to dream of ever ending the HIV-AIDS pandemic, at least, not by 2030 in LMICs.

## Competing interests

The authors declare no competing interests.
